# Adherence and acceptability of community‐based distribution of micronutrient powders in Southern Mali

**DOI:** 10.1111/mcn.12831

**Published:** 2019-10-17

**Authors:** Natalie Roschnik, Hawa Diarra, Yahia Dicko, Seybou Diarra, Isobel Stanley, Helen Moestue, Judy McClean, Hans Verhoef, Sian E. Clarke

**Affiliations:** ^1^ Programme Quality and Policy Save the Children UK London UK; ^2^ Sponsorship programmes Save the Children International Bamako Mali; ^3^ Department of Disease Control London School of Hygiene and Tropical Medicine London UK; ^4^ Department of Education and Child Protection Save the Children USA Washington DC; ^5^ Faculty of Land and Food Systems University of British Columbia Vancouver British Columbia Canada; ^6^ Medical Research Council Unit The Gambia at London School of Hygiene and Tropical Medicine London UK; ^7^ Cell Biology and Immunology Group Wageningen University Wageningen The Netherlands; ^8^ Division of Human Nutrition and Health Wageningen University Wageningen The Netherlands

**Keywords:** cluster randomised controlled trial, complementary feeding, community‐based, infant and child nutrition, malaria, Mali, micronutrients, preschool children

## Abstract

Home fortification with micronutrient powders (MNP) has been shown to reduce anaemia, with high overall acceptability and adherence, but there is limited evidence from West Africa. Around 80% of children younger than 5 years are anaemic in Mali, and new interventions are needed. This paper reports on the adherence and acceptability of a community‐led MNP intervention targeting children aged 6–59 months in Southern Mali. The MNP were delivered by a multidisciplinary group of community volunteers using community‐based preschools, cooking demonstrations, and traditional communication networks to promote MNP, nutrition, hygiene, and child stimulation. The MNP were delivered alongside early childhood development interventions and seasonal malaria chemoprevention. Adherence and acceptability were evaluated through two cross‐sectional surveys in 2014 and 2016 and a qualitative evaluation in 2015. Over 80% of parents reported ever having given MNP to their child, with 65% having given MNP for four or more days in the last week. Likely contributors to uptake include: perceived positive changes in the children following MNP use, the selection of a food vehicle that was already commonly given to children (morning porridge or bouillie) and the community driven, decentralized and integrated delivery approach. These findings support recommendations from recent reviews of MNP implementation to use community‐based delivery approaches and behaviour change components.

**Key messages**

- Community‐based and community‐led delivery of micronutrient powders (MNP) is feasible and can help achieve high uptake and acceptability.
- Choice of a food vehicle already given daily to individual children (such as morning porridge) promotes adherence and limits sharing.
- Combining MNP with seasonal malaria chemoprevention are complementary approaches to address the multifactorial causes of anaemia.
- The use of community preschools and multisectoral community volunteers offers a supporting environment and delivery mechanism for a range of complementary interventions to promote good nutrition practices and child development alongside MNP.

## 1 INTRODUCTION

Between 40% and 50% of children younger than 5 years in developing countries are iron deficient (UNICEF, 2003). In 2011, the World Health Organization (WHO) recommended home fortification of foods with micronutrient powders (MNP) to improve iron status and reduce anaemia among infants and children aged 6–23 months, where the prevalence of anaemia in children is 20% or higher (World Health Organization [WHO], 2011). The success of the home fortification, however, depends on caregivers' acceptability and adherence to the MNP treatment protocol and children consuming the recommended amount of MNP. Recent systematic reviews of MNP trials in low‐income countries have found that overall MNP reduced anaemia (De‐Regil, Jefferds, & Pena‐Rosas, 2017; De‐Regil, Suchdev, Vist, Walleser, & Pena‐Rosas, 2013), acceptability was high, and adherence ranged from 50% to 90% (De Barros & Cardoso, 2016). However, there is limited evidence on MNP adherence and impact from West Africa and none from Mali, where 80% of children younger than 5 years are anaemic (European Commission, 2017). There is also limited evidence of MNP effectiveness in contexts where the burden of malaria is high. In Mali, 52% of children aged 6–59 months are infected with malaria, 62% in Sikasso region (Ministère de la Santé Mali, 2010).

In 2013, a cluster‐randomised trial was launched in 60 rural villages in Sikasso and Yorosso districts in Southern Mali to evaluate the impact of home fortification with MNP, combined with seasonal malaria chemoprevention (SMC; WHO, 2012) and early childhood development (ECD) interventions, on children's health, nutritional status, and development. This paper summarises findings relating to implementation of the MNP intervention, MNP adherence, and acceptability, assessed through a series of qualitative studies and quantitative surveys conducted during the trial period 2013–2016.

## 2 METHODS

### 2.1 Overall trial design

A cluster‐randomised trial was conducted in 60 rural communities with Save the Children‐supported preschools in the districts of Sikasso and Yorosso in the Sikasso region in Southern Mali. The 60 communities were selected from a longer list of 75 communities with Save the Children‐supported preschools, prioritising those with the longest time since establishment. The 60 communities were randomly allocated to the intervention (with MNP distribution) or control group (no MNP distribution) in 2013. The intervention group received MNP delivered through the organising mechanisms of the community‐based preschool for three consecutive years. All communities (MNP intervention and control) also received SMC to reduce malaria incidence and, from October 2015, parenting education focusing on infant young child feeding, hygiene, cognitive stimulation, and child protection. Qualitative formative research was undertaken in 2013 to help design the MNP intervention, and in 2014, after the first year of MNP distribution, a second qualitative study was conducted to identify any need for course correction. Two quantitative surveys were conducted in 2014 and 2016 to evaluate the impact of MNP on stunting, anaemia, and child development, and data on MNP coverage and acceptability were recorded in parent questionnaires.

### 2.2 The intervention

#### 2.2.1 MNP protocol

All children aged 6–59 months in the 30 MNP communities were targeted to receive a sachet of MNP daily for four consecutive months, between January and April following SMC. This is the dry season when malaria transmission is at its lowest, before the rains and food shortages become more common. The WHO recommends 90 MNP sachets containing 10–12.5 mg of elemental iron to be given to children aged 6–23 months over a 6‐month period (WHO, 2011). Under this project, 120 MNP sachets containing 10 mg of iron were given to each child over a shorter 4‐month period to avoid provision of iron‐containing supplements during the malaria transmission season (which ends in December and starts again in May–June). Given the high prevalence of anaemia in the study area, the increased number of MNP sachets given (daily), coupled with SMC, this 4‐month daily MNP regimen was expected to markedly reduce the prevalence of iron deficiency anaemia. The MNP contained 400 μg of vitamin A, 5 μg of vitamin D, 5 mg of vitamin E, 0.5 mg of vitamins B1, B2, and B6, 0.9 μg of vitamin B12, 6 mg of niacinamide (vitamin B3), 150 μg of folate, 30 mg of vitamin C, 10 mg of iron (as encapsulated ferrous fumarate), 4.1 mg of zinc, 0.56 mg of copper, 17 μg of selenium, and 90 μg of iodine.

#### 2.2.2 Community‐based platform

Multisectoral community groups of volunteers, called Groupes de Soutien aux Activites Nutrition (GSAN), were created to deliver the MNP to caregivers at village level. They are composed of preschool teachers, the community midwife, the community health agent, women leaders, and two committed men (approximately eight people in total). GSAN members were trained by a regional team of Nutrition and ECD experts (government representatives) who themselves were trained by Nutrition and ECD experts from Save the Children and the Ministry of Health and Education at national level.

#### 2.2.3 MNP training

The MNP training was developed based on the findings and recommendations of the formative research, and drawing on national infant and young child feeding and parenting education guidelines and MNP materials from other countries. The training manual focused on diversifying the types of complementary foods given to young children and adding MNP to a range of foods (pureed fruit, mashed potato, enriched porridge, etc.). It also focused on play and child stimulation and essential hygiene practices to improve caregiving practices and child development. Job aids were created in 2015 to help GSAN members organise a cooking demonstration integrating nutrition, cognitive stimulation, and hygiene activities and remember key messages (Data S1 and S2).

#### 2.2.4 MNP implementation

Three consecutive distributions of MNP were organised in 2014, 2015 and 2016, reaching a total of 10,861 children in 2014 and 11,741 in 2016. Children received 4 months of daily MNP in 2014 and 2016 but only one round in 2015 due to a delay in MNP supply. GSAN members and village leaders first mobilised caregivers using informal village communication methods (networks of mothers, vaccination days, postnatal consultations, village fairs, and other gatherings), then organised cooking demonstrations to train mothers to prepare nutritious meals using locally available foods and add in the MNP. The distributions were organised neighbourhood by neighbourhood over 2–3 days depending on the size of the community. A special emphasis was put on ensuring that the child consumed the whole sachet by giving it to him with a small amount of food in his own cup. The mother practiced adding the MNP to the food prepared at the cooking demonstration and was given a box of 30 sachets of MNP (1 month's worth) for each child. The MNP were then added to children's meals by the main caregiver every day. A new box of 30 sachets was given to the mother every month on distribution days, and empty sachets were collected.

#### 2.2.5 Integration with SMC and ECD

**Figure 1:**
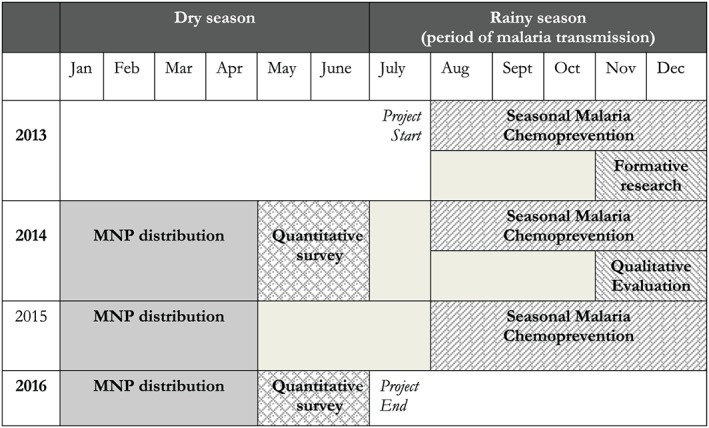
Timing of the micronutrient powders (MNP) and seasonal malaria chemoprevention (SMC) distributions and evaluation activities

Interventions that improve iron status can increase the incidence of infectious diseases, including malaria (Prentice et al., 2017), and MNP are not recommended in sub‐Saharan Africa except where malaria is well controlled (WHO, 2011). Thus, to minimise this risk, MNP were only distributed during the dry season for 4 months per year. Additionally, between August and December for three consecutive years (2013–2015) prior to each MNP distribution, all children aged 3–59 months in the target communities received SMC to reduce malaria‐related anaemia. The MNP and SMC were provided within the context of an existing Save the Children‐supported ECD programme—community preschools enrolling children aged 3–5 years and parenting education sessions open to all caregivers of children aged 0–5 years in the community. Figure 1 shows the timing of the MNP and SMC distributions in relation to the dry and rainy seasons and the qualitative and quantitative surveys.

### 2.3 Evaluation methods

The acceptability and adherence to MNP were assessed through qualitative and quantitative surveys conducted between 2013 and 2016.

#### 2.3.1 Formative research

In December 2013, a formative research study was conducted to inform the design of the MNP intervention. The research was conducted in four villages selected to represent the different geographic, socio‐economic, climatic, and market access conditions in the study area. In‐depth interviews were conducted with 22 community stakeholders (preschool teacher, local midwife, woman leader, health agent, community leader, and member of the school management committee) and 24 mothers, fathers, and grandmothers of children aged 6–59 months (eight of each type of caregiver); direct observations of mothers were conducted with four mothers and eight focus group discussions with mothers and fathers (four with mothers and four with fathers, a total of 56 people). All respondents provided informed verbal consent prior to the interview. Responses were recorded and transcribed in French and later translated and compiled in English textual data for iterative content analysis.

#### 2.3.2 Quantitative surveys

Two quantitative surveys were conducted in May–June of 2014 and 2016 in the 60 study communities. Both surveys included biomedical and child development assessments in children, described elsewhere (Save the Children, 2017), and interviews with their parent/caregiver. Health status and child development milestones were assessed in two groups of children, at age 3 years and at age 5 years, to represent the age at which children might enter and leave preschool, respectively. Sample size for the surveys was calculated to provide 80% power to detect a 26% reduction in anaemia (the primary outcome of the main trial) at 5% statistical significance for a cluster‐randomised trial with 30 communities per arm, assuming a conservative prevalence of 50% in the control group and an interclass correlation of 0.08. The minimum sample size was 20 children per community in each age group. Children aged either 1 year, 3 years, or 5 years in 2014 (20 per age group) were randomly selected from each community using a list of 3‐ to 59‐month‐old children resident in the village in the August 2013 census. Additional children were recruited in 2016 to compensate for losses to follow‐up primarily due to outmigration; these children were also randomly selected from the 2013 list to ensure that all children surveyed were eligible to have received the MNP intervention for three consecutive years. In both 2014 and 2016, a structured parental questionnaire was administered to the main caregiver of children aged 3 or 5 years to capture data on educational and socio‐economic backgrounds, home literacy environment, and MNP adherence and acceptability—a total of 1,200 interviews in the 30 MNP intervention villages in each survey round.

The data were analysed using STATA (version 14). Dietary diversity was estimated from parental report of the food groups eaten by a child in the previous day, with children eating less than or equal to four food groups classified as not meeting minimum dietary diversity (WHO, 2008). Independent determinants of MNP use were identified using mixed‐effects logistic regression with random effects by village using a backward modelling approach. All variables in the bivariate analysis where *P* value was <0.05 were included in the basic model. Multicollinearity was assessed using a correlation matrix of coefficients.

#### 2.3.3 Qualitative evaluation

A qualitative evaluation was conducted in December 2015 in 10 of the 30 MNP intervention communities selected to represent high and low MNP adherence (five of each) based on reported use of MNP in the 2014 quantitative survey and Save the Children field agents' own assessment of community and mother acceptability of MNP. The evaluation included 20 focus group discussions—10 with mothers and 10 with fathers of children aged 12 to 59 months, a total of 59 mothers and 60 fathers; in‐depth interviews with 30 mothers, 32 community leaders, and 24 GSAN members; and 10 direct observations of MNP use at household level. Semistructured discussion guides for each target group were developed and pretested in one village and administered in the local language by two trained enumerators. Responses were recorded directly on the interview guides and also tape recorded. The enumerators cross‐checked and completed their notes daily using the taped recording. The responses were then entered by trained data entry agents and cleaned and analysed by the research team using SPSS 2013 (version 22.0. Armonk, NY:IBM Corp), and a Memos system was used to identify key themes.

### 2.4 Ethical considerations

Ethical approval for (a) the trial and (b) follow‐up after 3 years of implementation was obtained from the Comite Ethique de l'INRSP, Ministry of Health, Mali [06/13/INRSP‐CE); 06/13/INRSP‐CE respectively] and LSHTM ethics committee, UK [6489; 11335]. In July 2013, community meetings were held with parents and local community representatives to explain the purpose of the study and procedures to be followed (including randomisation of communities), after which communities were offered the choice to participate in the trial. Community meetings were repeated in May 2014 and 2016 to obtain informed consent from the parents of each child selected to participate in the surveys. Signed consent prior to interview was also obtained from all individuals interviewed during the formative research and qualitative evaluation. To safeguard child rights, all project staff and survey team members were oriented on, and signed, Save the Children's child safety policy.

## 3 RESULTS

A summary of the various qualitative and quantitative research activities, characteristics of participants, and key findings is provided in Table 1.

**Table 1:** Summary of research methods and findings

	Pre‐intervention	Midterm evaluation (after 1 year of implementation)		Final evaluation (after 3 years of implementation)
	Formative research (December 2013)	Quantitative evaluation (May–June 2014)	Qualitative evaluation (December 2015)	Quantitative evaluation (may–June 2016)
Methods	‐IDIs ‐FGDs ‐Direct observations	‐Structured questionnaire with parent/guardian on reported MNP use and acceptability	‐IDIs ‐FGDs ‐Direct observations	‐Structured questionnaire with parent/guardian on reported MNP use and acceptability
Description of sample	‐Four villages ‐IDI with 22 community leaders and 24 caregivers (eight mothers, eight fathers, and eight grandmothers) ‐Eight FGDs (four with mothers and four with fathers): total participants = 56 ‐Direct observations in four households	‐All 60 villages (30 intervention and 30 control) ‐Random sample of 40 children per village (20 aged 3 years and 20 aged 5 years) ‐Total sample in intervention villages: 1,072 children but only 453 with complete data (of which 51% were boys, 49% girls; 99.6% aged 3 years, and 0.4% aged 5 years [due to loss of data])	‐10 villages (high and low adherence) ‐IDIs with 32 community leaders, 24 MNP distributors, and 30 mothers ‐20 FGDs: total participants 59 mothers and 60 fathers ‐Direct observations in 10 households	‐All 60 villages (30 intervention and 30 control) ‐Random sample of 40 children per village (20 aged 3 years and 20 aged 5 years) ‐Total sample in intervention villages: 1,148 children (of which 50% were boys, 50% girls; 49.5% aged 3 years; and 50.5% aged 5 years)
Findings	Opportunities and risks for MNP uptake: ‐Morning porridge *(bouillie*) had good potential as food vehicle for MNP because given routinely and pre‐existing tradition of enriching it to prevent anaemia ‐Disadvantages: The porridge is often liquid, thin, and hot, which could prevent MNP from mixing well with food and affect adherence ‐Tradition of family members eating out of the same bowl could lead to MNP being shared among several individuals. ‐Morning porridge is a meal served as individual portions, can thus target intended beneficiaries	Adherence, food vehicle, acceptability: ‐82% parents reported ever having given MNP to their child ‐65% reported giving MNP at least four times in the previous week ‐96% of MNP users reported adding MNP to the child's morning *bouillie* (liquid porridge), 21% to *tô* (solid porridge), and 11% to drinks or liquids ‐58% of users rarely/never having difficulties giving MNP to their child ‐91% noticed changes in their child since giving MNP, of whom 97% noticed positive changes ‐98% said they wanted to give MNP to their child the following year	Delivery approach, food vehicle, and acceptability: ‐Preschool infrastructure and cooking demonstration considered an effective delivery platform but awareness raising using a wider range of channels was recommended ‐Good overall understanding of how to administer MNP ‐Preference for daily MNP regimen (vs three to four times per week) ‐Morning *bouillie* preferred food vehicle because already given to child on a daily basis ‐MNP generally found to be easy to give and perceived to have positive impact (most noticed positive changes in their children)	Adherence, food vehicle, acceptability: ‐78% parents reported ever having given MNP to their child ‐66% reported giving MNP at least four times in the previous week ‐92% of users reported adding MNP to the child's morning *bouilllie*, 4% to *tô* (solid porridge), and 0.4% to drinks/liquids ‐92% of MNP users reported rarely/never having difficulties giving MNP to their child ‐96% noticed changes in their child, of whom 86% noticed positive changes ‐98% said they wanted to give MNP to their child again the following year

Abbreviations: FGDs, focus group discussions; IDIs, in‐depth interviews; MNP, micronutrient powders.

### 3.1 Formative research

The formative research found that the complementary foods most commonly provided to young children were a local bouillie (a thin, liquid, hot porridge, usually given to young children in the morning) and tô (a solid, congealed porridge) both made from maize or millet. Neither were considered appropriate food vehicles for MNP because they were either too liquid, too hot (*bouillie*), or too hard (*tô*). MNP need to be mixed into a semisolid and cool food to prevent the lipid‐encapsulated MNP from floating or sticking to the side of containers (reducing the amount ingested by the child) or the MNP melting in hot foods, exposing the elemental form of iron, changing the taste, colour, or odour of the food, which could reduce acceptability and adherence. Other meals were often eaten from the same bowl or plate as other family members (increasing the risk of sharing the MNP with other family members) and prepared by different family members each day, and feeding support or encouragement was rare. On the other hand, the large support group for mothers to assist each other in feeding and giving MNP to children, the existing awareness of anaemia, and the pre‐existing practice of “enriching” *bouillie* to prevent anaemia and malnutrition by mixing in dried bean powder were identified as opportunities for successful MNP uptake.

### 3.2 Qualitative evaluation after first year of implementation

#### 3.2.1 Sample characteristics

A total of 60 fathers and 59 mothers participated in 20 focus group discussions. In‐depth interviews were conducted with 30 mothers, 24 members of village GSAN committee (nine preschool staff, four traditional midwives, four community health agents, and seven women leaders), and 32 community leaders (10 village chiefs, nine religious leaders, five school directors, one school management committee member, three women leaders, two ECD management committee leaders, one mayor, and one traditional healer).

#### 3.2.2 MNP delivery approach

In‐depth interviews and focus group discussions revealed that most parents appreciated the preschool as a platform for MNP delivery and felt the preschool staff were the best people to provide information related to MNP: “The most credible source of information is the Monitrice (preschool staff),” said one mother, although almost half the men and some women also recommended the village chief's house for logistical reasons: “At the village chief's house, that is where there is the most space.” To maintain high MNP acceptability and support, mothers recommended multiplying the awareness‐raising platforms through all types of gatherings (such as preschool meetings, mothers' group weekly meetings, village authority meetings, vaccination days, the mosque on Fridays, family and youth meetings, trainings and educational discussions at the health centre, cell phone messaging, and leaflets); using traditional village hierarchy, starting with the village chief and his advisors, who then inform household heads and presidents of community associations, who in turn transfer the messages to families; going door‐to‐door to invite household heads (fathers); and involving school teachers, women leaders, and the village caller. Almost all mothers felt they had received the information they needed to administer the MNP to their child, and when asked what additional information they needed, responses ranged from “where can we find MNP if we need them?” to “what do the MNP contain?”, though the most common was around preparing nutritious meals. The main concern of mothers, fathers, village authorities, and GSAN members related to training: “We also want to be trained and receive information about MNP,” said one village Chief; “I also want to be included in the village nutrition activities,” said a traditional healer. The second concern was if and how they would continue receiving MNP. The frequency of cooking demonstrations varied by community. Some GSAN members reported never doing a cooking demonstration, others doing it once per month, and some up to five times per month. The main reason for not doing cooking demonstrations was lack of ingredients to prepare the meal (which had to be provided by the community) or lack of organisation and incentive (e.g. financial or in kind).

#### 3.2.3 MNP administration and food vehicle

Overall, everyone (men and women) knew about MNP, and all mothers knew the MNP protocol (one sachet per child per day). Both men and women preferred the more rigid daily MNP regimen (rather than three to four times per week) because it was easier to remember. Most mothers could name the key administration steps, but only a third mentioned that MNP should be added to semihard, soft pureed food. All mentioned the *bouillie* as the main food vehicle for MNP. For all mothers except two, morning breakfast was the best time to give the child the MNP. Other food vehicles mentioned included green leaves, rice, beans, *tô*, and potato puree, with some mentioning coffee (when they had no food). Most mothers said their children drank the MNP mix from a cup, others ate it with a spoon, and a small minority ate it with their hands. All mothers described the food to which the MNP were added as either cold or warm (i.e. not hot).

#### 3.2.4 MNP acceptability

Almost all mothers interviewed had found it easy to give the MNP to their child. Just under half of the women said they noticed a change in the food after the MNP was mixed in—a change in colour to yellow/orange or brown/dark or in taste—but the children did not react to these changes. Almost all mothers interviewed noticed a change in their child after taking the MNP. In focus group discussions, mothers and fathers often said their child was “healthier, more active,” “growing faster, more dynamic,” and “had more appetite, was more turbulent and their illness improved.” Both mothers and fathers said these perceived positive effects encouraged them to continue giving MNP. Negative effects mentioned were few and included diarrhoea; the child became ill, constipated, or got nausea/vomiting; and the child's stools became black. Most mothers said that their child accepted the food with MNP, with some mothers calling it “powdered milk” to encourage children to eat the food. All mothers said they wanted their child to receive the MNP the following year. No one in the community or family openly objected to the use of MNP, although there was one report (by GSAN members) of sachets being emptied on the floor when the child refused to consume them so that the mother could still return the empty sachets.

### 3.3 Quantitative surveys of reported MNP use

Findings from the questionnaire surveys with parents carried out immediately after the first round of MNP distribution (May–June 2014) and repeated 2 years later (May–June 2016) in the 30 intervention communities are summarised in Table 2.

**Table 2:** Caregiver reports on MNP use and acceptability from questionnaire surveys in 2014 and 2016

Indicators	2014 surveys (after 1 year of MNP distribution)		2016 surveys (after 3 years of MNP distribution)	
MNP coverage	***n*/*N***	**%**	***n*/*N***	**%**
Caregivers said they have ever added MNP to their child's food	369/453	81.5	899/1,148	78.3
Caregivers said they added MNP to their child's food at least 4 days per week in the last 7 days	295/453	65.1	755/1,148	65.8
MNP vehicle used				
Liquid porridge (*bouillie*)	336/350	96.0	847/891	95.1
Bean puree	10/350	2.9	94/891	10.5
Drinks	40/350	11.4	69/891	7.7
Solid congealed porridge (*tô*)	74/350	21.1	36/891	4.0
Other^a^	30/350	8.6	152/891	17.1
MNP acceptability				
Children liked to eat the food with the MNP	N/A	N/A	857/908	94.4
Caregiver rarely or never had difficulties giving MNP to their child	187/323	57.9	835/904	92.4
Parents noticed changes in their child since giving MNP	264/290	91.0	824/855	96.4
Parents noticed one or more positive changes in their child:	255/264	96.6	705/823	85.7
Child appetite increased	199/264	75.4	537/823	65.2
Child less sick than normal	162/264	61.4	407/823	49.4
Child more active/energetic/turbulent	201/264	76.1	327/823	39.7
Parents noticed one or more negative changes in their child	39/264	14.8	118/823	14.3
Child more sick than normal	16/264	6.1	46/823	5.6
Child less active/energetic than normal	25/264	9.5	39/823	4.7
Parents who want to give MNP the following year	335/341	98.2	871/890	97.9

Abbreviation: MNP, micronutrient powders.

a Other foods include: coffee (4%; 14/350), sauce (4%; 13/350), and rice (0.9%; 3/350) for 2014; coffee (4%; 37/891), sauce (5%; 40/891), rice (2%; 18/891), fruits (banana, papaya, and mango; 0.7%; 7/891), and vegetables (green beans, potato; 3%; 28/891) for 2016.

#### 3.3.1 Sample characteristics

A total of 1,072 children and their caregivers were surveyed in 2014, and 1,148 caregivers were surveyed in 2016. Unfortunately, approximately half the data on MNP adherence and acceptability from 2014 were lost due to a computer virus. The remaining usable data represent 42% of the total sample (453 out of 1,072), from all 30 villages in variable proportions (23% to 67%), and equal numbers of boys and girls (43% of boys and 42% girls). However, almost all the 5‐year‐olds' data and 18% of the 3‐year‐olds' data were lost. Data obtained in 2016 were more representative. Of the 1,148 caregivers (usually mothers) interviewed in 2016, 579 were parents of 5‐year‐old children, and 568 were parents of 3‐year‐olds, with an equal number of girl and boy children (570 boys and 571 girls). The main languages spoken in the households were Shenara (49%) and Bambara (35%); 79% of fathers and 82% of mothers had never attended school, and the main source of livelihood was subsistence agriculture (94%). 55% of 5‐year‐old and 41% of 3‐year‐old children were attending the preschool at the time of survey.

#### 3.3.2 MNP coverage

The intervention achieved good coverage in both 2014 and 2016, with 81.5% and 78.3% of caregivers reporting that they had ever given their child MNP (Table 2). When asked about use in the previous week, 65% said they had given MNP to their child four or more times in the last 7 days. Although reported use of MNP in 2016 exceeded 80% in more than half of the villages (17 of 30), it did vary between communities (from 38% to 97%), suggesting challenges in certain communities. However, only two villages had an adherence of less than 60% (one with 38% and another with 50%), and seven villages had an adherence between 60% and 70%.Table 3 compares characteristics between MNP users and nonusers. Results from this multivariate regression analyses revealed significant associations between the quality of a child's diet and MNP use. Children whose diet met minimum dietary diversity were 1.82 times more likely to have received MNP than children who had poor dietary diversity (OR:1.82, 95%CI [1.28, 2.61], *P* < 0.001); similarly, MNP use was less common in children whose parents reported that the variety of foods in their diet was limited (OR:0.65, 95%CI [0.46, 0.93], *P* = 0.02).

**Table 3:** Characteristics of households in relation to reported MNP use (ever/never) in 2016 (*n* = 1.110)^a^ ^a^

Characteristics	Reported ever use of MNP (*n* = 890)	Reported MNP never used (*n* = 220)	Basic model (OR 95% CI)		*P*	Adjusted model *(OR 95% CI)*		*P*
Age group					0.41			0.90
3 years	81.0% (440/543)	19.0% (103/543)	1			1		
5 years	79.3% (450/567)	20.6% (117/567)	0.87	0.64,1.20		0.98	0.70,1.37	
Sex					0.48			0.45
Male	80.8% (445/551)	19.2% (106/551)	1			1		
Female	79.4% (439/553)	20.6% (114/551)	0.89	0.64,1.23		0.88	0.63,1.23	
Ethnicity					0.42	Excluded		
Bambara	79.1% (307/388)	20.9% (81/388)	1					
Shenera	80.2% (450/560)	19.8% (111/560)	0.87	0.51,1.51				
Mamara	87.0% (60/69)	13.0% (9/69)	1.90	0.66,5.44				
Other	80.0% (74/93)	20.4% (19/93)	0.72	0.33,1.56				
Wealth index					0.22			0.33
Poorest^b^	76.8% (298/388)	23.2% (90/388)	1			1		
Least poor	82.2% (578/703)	17.7% (125/703)	1.25	0.75,2.09		1.20	0.83,1.72	
Father ever attended school					0.02			0.09
No	78.1% (677/867)	21.9% (190/867)	1			1		
Yes	88.3% (203/230)	11.7% (27/230)	1.77	1.10,2.80		1.50	0.93,2.42	
Mother ever attended school					0.03			0.07
No	79.0% (708/897)	21.1% (189/897)	1			1		
Yes	86.0% (178/207)	14.0% (29/207)	1.67	1.06,2.61		1.53	0.95,2.47	
Minimum dietary diversity of child's diet in previous day^c^					<0.001			0.001
Does not meet minimum	72.6% (291/400)	27.3% (109/400)	1			1		
Meets minimum	84.4% (599/710)	15.6% (111/710)	1.99	1.43,2.80		1.82	1.28,2.61	
Did child eat limited variety of foods in the last 4 weeks					<0.001			0.02
No	83.4% (559/670)	16.6% (111/670)	1			1		
Yes	74.8% (314/420)	25.2% (106/420)	0.58	0.42,0.82		0.65	0.46,0.93	
Did child go to sleep hungry due to lack of food in past 4 weeks					0.64	Excluded		
No	80.2% (817/1,019)	19.8% (202/1,019)	1					
Yes	80.9% (68/84)	19.1% (16/84)	0.86	0.46, 1.61				
How many meals or snacks did child get yesterday					0.11	Excluded		
1–2	92.9% (26/28)	7.1% (2/28)	1					
3	77.6% (260/335)	22.4% (75/335)	0.24	0.05,1.08				
4	77.8% (284/365	22.2% (81/365)	0.25	0.05,1.13				
5	83.8% (320/382)	16.2% (62/382)	0.33	0.07,1.54				

*Note*. Basic model is fixed effects logistic regression model with random effects by village. *P* values derived using likelihood ratio tests.

Abbreviations: 95% CI, 95% confidence interval; MNP, micronutrient powders; OR, odds ratio.

a Analysis excludes 37 interviews with missing data on one or more of the explanatory variables.

b Lowest quartile. Wealth score calculated using principle components analysis of reported ownership of households assets.

c Minimum dietary diversity: Score based on number of food groups eaten by child in the previous day, children eating less than or equal to four food groups classified as not meeting minimum dietary diversity (World Health Organization, 2008).

#### 3.3.3 MNP vehicle

The most common food vehicle for the MNP was the *bouillie* (mentioned by over 95% of caregivers in both 2014 and 2016). Other food vehicles used in 2016 were bean puree (10.5%), drinks (7.7%), and *tô* (4.0%). Compared with that in 2014, the reported practice of adding MNP to bean puree had increased, whereas use of less suitable vehicles such as drinks and *tô* had decreased.

#### 3.3.4 MNP Acceptability

Most parents who reported giving MNP to their child found them easy to administer; after the first year of implementation, 58% of caregivers reported rarely or never having any difficulties giving MNP to their child. By the third year, this was 92%. Most parents reported noticing changes in their child since giving them the MNP. In 2016, 86% reported noticing one or more positive changes (increased appetite, less sickness, and more activity), and only 14% mentioned one or more negative changes (child is sicker or less active). Over 94% of the parents reported that their child liked the food with the MNP added, and 98% of parents said they would like to give their child MNP again.

## 4 DISCUSSION

MNP have the potential to reduce anaemia prevalence in young children. This study in a poor and rural population in Southern Mali demonstrates good levels of adherence and acceptability of the intervention, with over 65% of caregivers reporting giving their child MNP for four of more days in the previous week and 98% wanting to give MNP again in the following year. The findings on MNP acceptability and adherence are consistent across the various qualitative and quantitative studies conducted during the trial period and compare favourably with similar studies (De Barros & Cardoso, 2016; Reerink et al, 2017).

Several factors are likely to have contributed to the generally high levels of adherence and acceptability. The first is the high proportion of caregivers that perceived positive changes in their children, which encouraged mothers and fathers to give MNP to their child. The second is the food vehicle selected, the morning *bouillie*, reported to have been used by almost all caregivers (95%). The daily routine of preparing and giving the morning porridge helped mothers remember to add the MNP. A further advantage of this food vehicle in a society where meals are communal, with children usually eating from a common bowl, was that it enabled MNP to be targeted to individual children (in a cup) and the full dose to be consumed. Some disadvantages of the morning *bouillie* is that the MNP may stick to the side of the cup if it is too liquid, disintegrate if too hot, or discourage mothers of making their *bouillie* more nutritious by adding other ingredients, thinking that the MNP provides all the nutrients the child needs. All home fortification strategies also rely on foods being available to which the MNP can be added. Children in households who did not use MNP had a lower dietary diversity, and parents more frequently reported that the child's diet was limited by a lack of resources. From the data available, we are unable to distinguish whether this difference was due to an improvement in dietary diversity, as well as MNP use, among parents who engaged with the intervention, or whether a lack of food hindered the regular use of MNP. Either way, this finding signals a concern that MNP may not reach those children that might need it most.

We believe the delivery approach facilitated strong community support for the intervention. We used a community‐based and integrated approach, relying on multisectoral community volunteers as MNP distributors (GSAN), along with traditional communication networks and cooking demonstrations to promote child stimulation and appropriate infant and young child feeding and hygiene practices alongside MNP. Moreover, the MNP delivery was conducted jointly with the delivery of two popular and ongoing malaria and ECD interventions that mobilised large numbers of mothers and children. Community preschools have also been successfully used as a delivery platform in previous research, showing the potential of involving preschool and primary‐school structures and staff for health interventions (Gelli et al., 2018). Other factors include the long‐term presence of Save the Children in the area and high levels of trust with communities, as well as a regular supply of MNP, which were transported directly by Save the Children to the preschools. Adherence, however, did vary between communities (from 38% to 97%), suggesting some differential uptake, acceptability, and/or variation in implementation between communities.

Strengths of our study lie in the large sample size, mixed methods approach, and longer intervention period. Despite partial data loss in 2014 due to a computer virus, there is no evidence that this introduced any data bias, because the survey results from 2014 and 2016 were remarkably similar. Nonetheless, we believe the quantitative data from 2016 provide a better indicator of acceptability of MNP, being measured after three consecutive years of experience with the intervention. The sample size for the quantitative survey was large compared with those in other MNP studies (De Barros & Cardoso, 2016) and is likely representative of the target community, whereas the mixed methods approach allowed for a more comprehensive assessment of adherence and acceptability. Consistency between the results obtained using different methodologies adds further credence to our findings. Other studies have also used either quantitative (Adu‐Afarwuah et al., 2008; Menon et al., 2007; Mirkovic et al., 2016) or qualitative (Jefferds et al., 2010; Tripp et al., 2011) methods to assess adherence and acceptability of MNP, but few have used a mixed methods approach. Finally, although other MNP intervention studies also supplied MNP for between 2 and 6 months per year, only a few carried out the distributions for more than 1 year. This allows for both growing familiarity with the intervention and continued improvements in nutritional status, building on the previous years' gains, to be assessed. Although disruption to MNP distribution in 2015 likely hindered this process, this is a problem commonly encountered by others too (Christofides, Schauer, Sharieff, & Zlotkin, 2005; Locks, Reerink, & Tucker Brown, 2017; Mirkovic et al., 2016). The primary limitation of our study is that although our results were consistent across the surveys and methods used, the data are largely based on parent reports of adherence and acceptability and could be subject to social desirability bias. Adherence to MNP interventions in many studies was also self‐reported (Adu‐Afarwuah et al., 2008; Christofides et al., 2005; Menon et al., 2007; Mirkovic et al., 2016), whereas others used sachet counts (Ip, Hyder, Haseen, Rahman, & Zlotkin, 2009; Jefferds et al., 2010). A study in Kenya comparing adherence using self‐reporting, sachet count, and an electronic monitoring device found that adherence is overestimated both by self‐reporting and sachet counts (Recrink et al., 2017). This risk is increased in the context of our study, where the intervention and evaluations were organised by communities' long‐term nongovernmental organisation partner, which they may be keen to please. However, the pre‐existing relationships, community infrastructure, and networks could also have served to genuinely enhance MNP acceptance, which may be less readily replicated elsewhere.

In conclusion, this study supports the findings and recommendations from recent reviews of MNP implementation, which identify similar factors that support higher MNP adherence. Factors promoting adherence include caregiver perception of positive changes and child acceptance of the food containing MNP, while forgetfulness was the main reason caregivers would not give MNP to their children. The implementation design features, such as communication channels, messages and administration regimen, also influenced caregiver knowledge and adherence. (De Barros & Cardoso, 2016; Reerink et al. 2017). This study also supports MNP guidelines that recommend a behaviour change strategy that promotes hygiene and healthy diets alongside MNP use (WHO, 2016). Recommendations for future MNP interventions are to use community‐based and community‐led approaches and to identify an appropriate food vehicle that caregivers are already giving to their child daily.

## CONFLICTS OF INTEREST

The authors declare that they have no conflicts of interest.

## CONTRIBUTIONS

The intervention was conceived, designed, and implemented by NR, HD, YD, and SD, with additional inputs from JM and SEC. NR, HD, YD, SD, HV, JM, and SEC contributed to the development of the evaluation tools and acquisition of the data. All authors contributed to the analysis and interpretation of the data, critical revision of the manuscript, and approval of the final version.

## Supporting information

Data S1. Supporting Information

Data S2. Supporting Information
