# Supplementary material for: Adherence and acceptability of community‐based distribution of micronutrient powders in Southern Mali
Source: Matern Child Nutr. 2019 Oct 17;15(Suppl 5):e12831. doi: 10.1111/mcn.12831 (PMC6856685; doi:10.1111/mcn.12831)
Supplement: Supplementary file 1 — Data S1. Supporting Information [file MCN-15-e12831-s001.docx]

**Supplementary material 1 (annex 1): Job aid to help GSAN members organise a cooking demonstration (see footer for translation*)**

*Translation:

Page 1:

- Title: Well nourished, clean, stimulated and vitamins
- Sub title: Job aid for Groupes de Soutien des Activites Nutritionnelles (GSAN)
- Grey box: Preparation; 1. Toys (objects from home and preschool); 2. Food ingredients: women bring; 3. Kitchen utensils: firewood, pan,…; 4. Micronutrient powders (MNP); 5. Advice cards, monitoring sheets
- Red title box: 10 steps for a cooking demonstration
- Vertical titles (left): Before cooking; preparing meal; during cooking
- Box 1: **Step 1: Welcome**; - **Objectives:** “We will learn how to feed stimulate and prevent illness for their health and education”; - ***Pretest***: questions on theme (yes/no with hand signs)
- Box 2: **Step 2: IYCF and anaemia**; - ***Demonstration***: with bags of 1000ml and 200ml; ***Questions***: consistency, Quantity, variety, active feeding; - ***Anaemia***: how to recognise it? Which foods prevent anaemia? ***Presentation of foods*** (demonstration) with the 5 stars
- Box 3: **Step 3: Cooking demonstration**; - ***Handwashing***: GSAN demonstrates and asks at which other times? 4 key times; Volunteers wash their hands too (before preparing meal); - ***Cooking demonstration***: preparation of a healthy and balanced meal that PMN should be mixed to; - ***Questions and explanation*** (during demonstration): - Variety: Insist on 3^rd^ (animal product) and 4^th^ stars (fruit/vegetable); - Hygiene: clean and fresh ingredients, clean work space and utensils
- Box 4: **Step 4: Play**; ***Questions***: What makes your child laugh? How does he learn? Play, talk, listen and encourage your child and he will learn to speak, read and write faster; - ***Demonstration and practice***: games with and without toys from the home. It’s pause time!
- Box 5: **Step 5: Sick child**. ***Questions***: what illnesses does your child often get? Can he grow and learn when he is sick? What to when he is sick? ***Demonstration***: bottle/gourd/bag with hole below to show dehydration and need for more liquids; ***Questions***: What else does a sick child need? More liquids, one more meal, encouragement and go to health centre
- Note: IYCF=Infant and Young Child Feeding

Page 2:

- Title: Well nourished, clean, stimulated + vitamins
- Vertical boxes (left): During cooking; We wash hands and we eat! After the meal
- Box 1: **Step 6: Vitamins (MNP**); - ***MNP presentation*** (while the meal is cooking), show the sachet and speak of the benefits for health (anaemia) and child development; - ***Utilisation***: 1 per day per child: shake the sachet before opening: add too food with thick consistency, soft or semi solid foods or puree (fruit, potatoes, pumpkin, etc): separate small quantity, mix and give before the rest; - ***Do not add to:*** To, coffee, tea, other hot foods or liquids, solid foods; ***side effects***: soft stools (4-5 days), temporary constipation, black stools; ***Sick child***: continue giving MNP, got to health center, MNP is not a medicine
- Box 2: **Step 7: Hand washing (hygiene)**; - ***Demonstration*** (by volunteers): Wash hands with soap – running water, rub, ashes OK; ***Questions***: At what other moments should one wash hands? 4 key moments: before preparing food, before eating, after latrines, after cleaning faeces – often! ***Practice*** (all mothers and their children): all wash their hands and their children’s hands
- Box 3: **Step 8: We eat and add MNP**; ***MNP distribution***: Serve the meal in bowls and while food cools on clean matt, distribute MNP to mothers; - ***Add MNP***: each mother adds MNP – separate and mix; ***We eat***: mothers taste the food mixed with MNP, give the food with MNP to children and encourage the child
- Box 4: **Step 9: Recap** (during quiet moment); - ***Discussion***: What did you learn today? What will you do at home? Remind basic concepts for IYCF, anaemia, hygiene, stimulation and MNP and adaptation to daily routine; ***Neighbours help each other***: women create groups of 4-5 neighbours/friends to help and support each other, with 1 GSAN member to supervise them; ***Promises***: in their small groups, they make promises “ I will talk with my child every day when I cook…”
- Box 5: **Step 10: Goodbye and music**; ***in music!*** Start a rhythm with objects/toys and encourage mothers and children to add a rhythm and song; ***create a ‘MNP’ song*** or on other themes of the day
