# Supplementary material for: Adherence and acceptability of community‐based distribution of micronutrient powders in Southern Mali
Source: Matern Child Nutr. 2019 Oct 17;15(Suppl 5):e12831. doi: 10.1111/mcn.12831 (PMC6856685; doi:10.1111/mcn.12831)
Supplement: Supplementary file 2 — Data S2. Supporting Information [file MCN-15-e12831-s002.docx]

**Supplementary material 2 (annex 2): Visual aid of key messages (see translation in Footer*)**

*Translation Visual aid of key messages:

Page 1: red heading: IYCF (infant Young Child Feeding) – Anemia - MNP (1) Eat often; (2) vary the foods; (3) Give rich and thick porridge; (4) add vitamins (MNP); (5) encourage to eat

Page 2: red headings: Hygiene, Stimulation, Sick child. (6) wash hands with soap; (7) put faeces in the latrines; (8) Play and talk with your child; (9) Give more to dring and eat – continue the vitamins (MNP) and take to the community health center
